# Sebaceous Carcinoma of the Breast in a Japanese Male with a *BRCA2* Pathogenic Variant: Report of an Exceedingly Rare Case and Review of the Literature

**DOI:** 10.70352/scrj.cr.24-00543

**Published:** 2025-01-31

**Authors:** Mamika Kuribayashi, Tadahiro Isono, Yuichi Satake, Yuta Yokochi, Tomoki Kawamura, Ryosuke Kishi, Ryohei Koreyasu, Konomi Sakyo, Takahiro Watanabe, Takeshi Ueda, Masashi Nozawa, Mitsuhiro Tachibana, Kei Tsukamoto, Kazuyasu Kamimura, Hidetoshi Wada

**Affiliations:** 1 Department of Surgery, Shimada General Medical Center, Shimada, Shizuoka, Japan; 2 Department of Diagnostic Pathology, Shimada General Medical Center, Shimada, Shizuoka, Japan; 3 Department of Diagnostic Radiology, Shimada General Medical Center, Shimada, Shizuoka, Japan

**Keywords:** beast, sebaceous carcinoma, *BRCA* pathogenic variant, male breast cancer, case report

## Abstract

**INTRODUCTION:**

Sebaceous carcinoma (SC) is a malignant neoplasm differentiated from the mature sebocyte and occurs mainly in the periorbital area. However, SC of the breast is extremely rare. We report a case of sebaceous breast carcinoma in a Japanese man with a *BRCA2* pathogenic variant.

**CASE PRESENTATION:**

A 77-year-old Japanese man had been aware of a mass in his right breast for about a year and had visited his previous physician for a follow-up. Over the next year, the mass grew, and the last doctor he visited referred him to our hospital for further examination and treatment. Physical examination revealed a palpable 3-cm-large mass of the right breast. There was no skin invasion, and core needle biopsy revealed invasive ductal carcinoma cT2N1M0 cStage IIB, estrogen receptor (+)/progesterone receptor (+)/HER2/*neu* (–)/Ki-67 labeling index: 27.8%. His daughter had a history of breast cancer, and he had a *BRCA2* pathogenic variant. The patient underwent a total right mastectomy and axillary lymph node dissection. Pathological diagnosis was primary SC of the breast, pT2N0M0, pStage IIA. His postoperative clinical course was good. Postoperatively, the patient received endocrine therapy and S-1 for 1 year and is currently receiving endocrine therapy alone. One year and 5 months have passed since the operation, with no recurrence or metastasis noted.

**CONCLUSIONS:**

The prognosis of SC of the breast has not been elucidated. As cases of SC with a *BRCA2* pathogenic variant are exceedingly rare, it will be necessary to continue accumulating cases in the future to understand this disease further. This research is essential to elucidating SC.

## Abbreviations

SC
: sebaceous carcinoma
*BRCA2*
: breast cancer susceptibility gene II
WHO
: world Health Organization
HER2
: human epidermal growth factor receptor 2

## INTRODUCTION

Sebaceous carcinoma (SC) is a malignant neoplasm differentiated from sebaceous gland cells. It occurs mainly in the periorbital area and is the world’s fourth most common eyelid malignancy. Generally speaking, SCs exhibit clinically aggressive behavior wherever they present.^1)^ By contrast, SC of the breast is very rare, with less than 30 cases having been reported. Van Bogaert and Maldague first reported subtypes of lipid-secreting carcinoma of the breast in 1977,^2)^ and Prescott et al. first reported a case of SC of the male breast in 1992.^3)^

Histologically, the World Health Organization (WHO) classification has defined SC. According to the previous WHO classification, primary SC of the breast had to show sebaceous differentiation in at least 50% of cells, and there should be no evidence of origin from cutaneous adnexal sebaceous glands.^4)^ In the current WHO classification, primary breast SC must show prominent sebaceous differentiation and originate within the mammary gland parenchyma, with no evidence of origin from cutaneous adnexal sebaceous glands.^5)^

There has only been 1 reported case of SC in the male breast^6)^ and 1 reported case of female SC caused by a breast cancer susceptibility gene II (*BRCA2*) pathogenic variant.^7)^ Here, we report an exceedingly rare case of SC of the breast in a Japanese male with a *BRCA2* pathogenic variant.

## CASE PRESENTATION

**Fig. 1:**
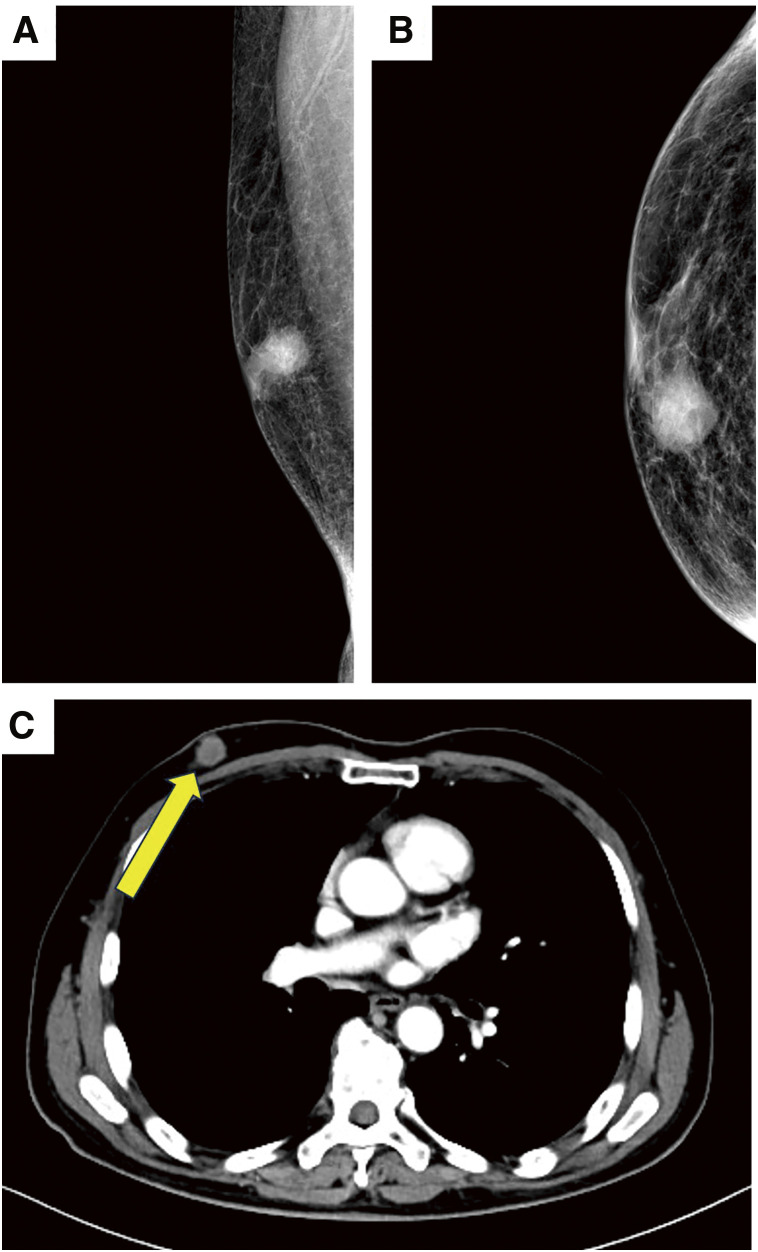
Mammogram results. Mammography shows a micro-lobulated and round mass without calcification in the right craniocaudal inner (**A**) and mediolateral-oblique middle areas (**B**). (**C**) Contrast-enhanced computed tomography reveals a solitary mass in the right breast (arrow).

A 77-year-old Japanese man had been aware of a mass in his right breast for about a year and had visited his previous physician for follow-up. Over the next year, the mass grew, and the last doctor he visited referred him to our hospital for further examination and treatment. Physical examination revealed a palpable 3-cm-large mass of the right breast with no skin findings. Laboratory data were within normal limits. There was no elevation in serum levels of any tumor marker, including CEA, CA15-3, and BCA225/CLEIA. A mammogram showed a micro-lobulated and round mass without calcification in the right craniocaudal inner (**Fig. 1A**), and mediolateral-oblique middle area (**Fig. 1B**). Contrast-enhanced computed tomography revealed a solitary mass in the right breast (**Fig. 1C**). The right axillary lymph node was enlarged. As a biopsy was not conducted for the right axillary lymph node swelling before surgery, metastasis was suspected based solely on computed tomographic imaging findings. Consequently, we classified the clinical stage as N1. There were no apparent distant metastases, and bone scintigraphy showed no apparent bone metastases. A mammary ultrasound revealed a 34 × 14 × 13-mm irregular mass in the areola to the upper inside area in the right breast. The patient’s daughter had a history of breast cancer, and his mother had a history of stomach cancer. He was tested for *BRCA1/2* pathogenic variants using BRACAnalysis CDx (Myriad Genetics, Salt Lake City, UT, USA), which showed that he had a *BRCA2* pathogenic variant.

A core needle biopsy was performed, which revealed invasive ductal carcinoma, solid type. The clinical staging was cT2N1M0: cStage IIB (TNM, UICC 8th edition). The patient underwent a total right mastectomy and axillary lymph node dissection. His postoperative clinical course was good, and he was discharged from our hospital on postoperative day 8.

**Fig. 2:**
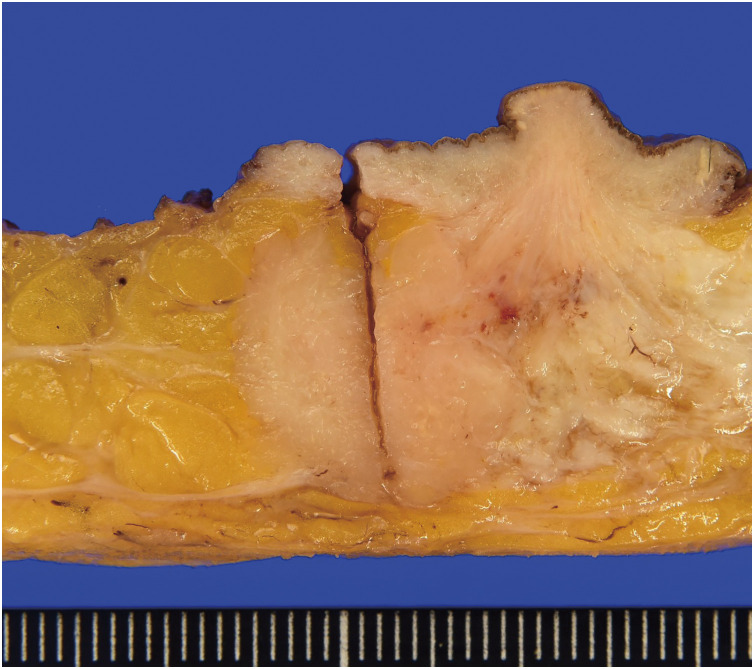
Photograph of the resected mass. Macroscopically, the tumor is 20 × 15 × 15 mm and has no connection to the overlying skin.

Macroscopically, the tumor was 20 × 15 × 15 mm in diameter, with well-defined borders and a milky-white cross-section. It had infiltrated the surrounding fatty tissue but was not connected to the skin (**Fig. 2**).

**Fig. 3:**
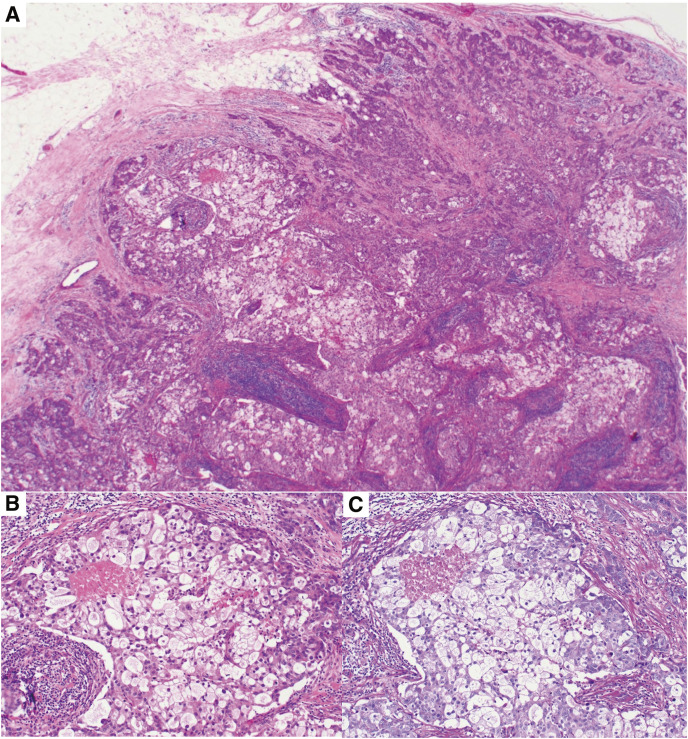
Photomicrographs of the mass. (**A**) The tumor includes cords, lobules, and solid sheets of cells with abundant vacuolated cytoplasm reminiscent of mature sebocytes (H&E staining, ×20). (**B**) The sebaceous cell component comprises 60% of all tumor volume. Foci of comedo necrosis in the sebaceous component are present in the solid nest (H&E staining, ×100). (**C**) No glycogen is found in the tumor cytoplasm (periodic acid-Schiff reaction, ×100).

Histologically, the tumor included cords, lobules, and solid sheets of neoplastic cells. The sebaceous carcinomatous component comprised 60% of all tumor volume (**Fig. 3B**). The neoplastic cells comprised abundant vacuolated clear cytoplasm reminiscent of mature sebocytes (**Fig. 3A**). The nuclei of the sebaceous element were relatively small and pyknotic, whereas the other nuclei were mainly small. Foci of comedo necrosis in the sebaceous component were also present (**Fig. 3B**). In the periodic acid-Schiff reaction specimen, no glycogen was found in the tumor cytoplasm (**Fig. 3C**). Squamous differentiation was not seen. Mitotic activity was more than 10 mitoses per 10 high-power fields. The stroma was densely collagenous and showed lymphoplasmacytic infiltration. The tumor-infiltrating lymphocyte score was calculated as 60%. Thin collagenous septa separated individual cords and small nests of tumor cells with a rich capillary network. None of the tumors reached the dermis nor showed pagetoid spread. The patient’s histologic grade was Bloom and Richardson grade III (tubule formation score 3, nuclear atypia score 3, mitotic counts score 2). There was no lymphovascular and perineural invasion and a lack of metastatic disease in the examined axillary lymph nodes. The pathologic staging was pT2N0M0: pStage IIA (TNM, UICC 8th edition).

**Fig. 4:**
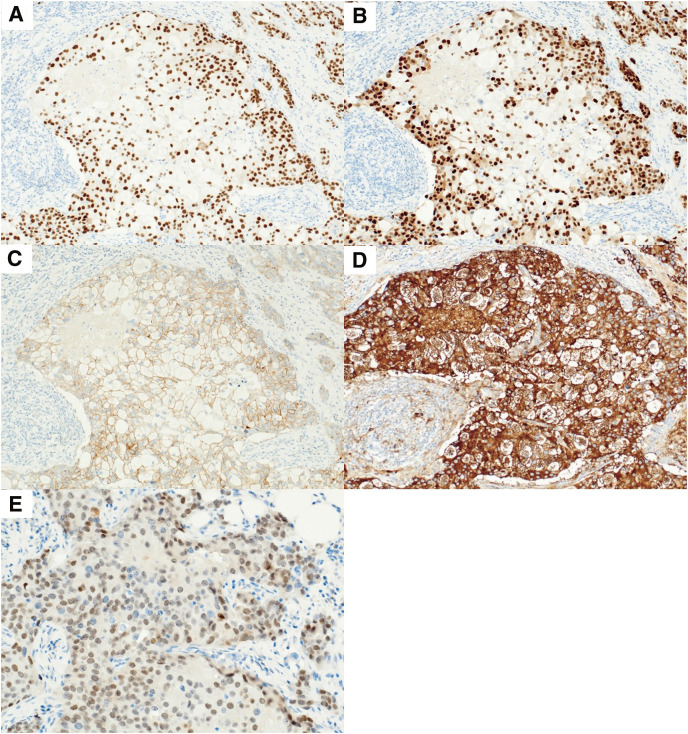
Immunohistochemistry results. (**A**) The tumor is positive for the estrogen receptor (×100) and (**B**) the progesterone receptor (×100). (**C**) The tumor is equivocally positive (Score: 2+) for HER2/neu (×100). (**D**) The tumor is positive for EMA (×100), and (**E**) the androgen receptor (×200).

Immunohistochemistry showed that the tumor cells expressed hormone receptors. Estrogen and progesterone receptors were expressed in 100% and 70% of the tumor cells, respectively (**Fig. 4A** and **4B**). Human epidermal growth factor receptor 2 (HER2)/*neu* (HercepTest; Agilent Technologies, Tokyo, Japan) was equivocally positive (score: 2+), and chromogenic *in situ* hybridization analysis (performed at an external laboratory) did not show amplification of the HER2/*neu* gene (**Fig. 4C**). The Ki-67 labeling index was 27.8%. p53 overexpression was not identified. GATA3, EMA, androgen receptor, and p63 were expressed, whereas cytokeratin 5/6 and adipophilin expression were negative (**Fig. 4D** and **4E**). Postoperatively, he received endocrine therapy and S-1 for 1 year and is currently receiving endocrine therapy alone. Given that the patient had estrogen receptor-positive, HER2/*neu*-negative breast cancer with histologic grade III, S-1 was administered in accordance with the POTENT trial protocol.^8)^ Although this was a clinical trial exclusively involving women, incorporating S-1 into endocrine therapy is strongly endorsed in the Japanese guidelines for patients with a high risk of recurrence.^9)^ One year and 5 months have passed since the operation, and no recurrence or metastasis has been noted.

## DISCUSSION

The authors experienced a rare case of SC of the breast in a Japanese male with a *BRCA2* pathogenic variant. The present tumor was classified as luminal B-like SC of the breast. There are 22 case reports of SC of the breast^2, 3, 10,11)^ in patients ranging in age from 25 to 85 years. The majority (7 cases) were luminal-like tumors (estrogen receptor (+)/progesterone receptor (+)/ HER2/*neu* (–)). In terms of outcome, most patients survive, and although there have also been reports of poor prognosis,^12)^ the outcome remains to be clarified. There has only been 1 reported case of female SC caused by a *BRCA2* pathogenic variant.^6)^ No cases of male-onset primary SC of the breast associated with *BRCA2* mutations have been reported. Furthermore, reported cases of SC of the breast in men are extremely rare, with a PubMed and Google Scholar search showing only 1 male case reported in 2018.^7)^ The WHO 5th Edition Blue Book hypothesizes that breast SC arises from the malignant transformation of local pluripotent cells with the capacity for divergent differentiation.^5)^ However, genetic studies remain insufficient, leaving the carcinogenesis of SC largely unknown.

In recent years, *BRCA* genetic testing has become a companion diagnostic test. The number of *BRCA* cases is expected to continue increasing, not only due to companion diagnostics but also because the criteria for HBOC testing have expanded to include factors such as family history and patient age. Therefore, although the relationship between *BRCA* pathogenic variants and SC is currently unknown, it may become apparent. In addition, knowing the *BRCA* pathogenic variant type may allow the use of olaparib. We have no plans to offer genetic counseling to our patient because he does not want it, nor do we plan to identify a *BRCA* pathogenic variant in third-degree relatives. A *BRCA2* mutation has been reported to be associated with an increased risk of breast, gastric, pancreatic, and prostatic cancer in men, and female carriers appear to be at higher risk of gastric cancer than male carriers.^13–15)^ Prostatic cancer screening is recommended for a man with a *BRCA2* pathogenic variant.^16–18)^ The present patient’s daughter had breast cancer. She was known to have a *BRCA2* pathogenic variant, so there may be a high correlation between this family’s cancer history and the *BRCA2* pathogenic variant. As our patient may develop prostatic cancer in the future, we recommended that he undergo prostatic cancer surveillance, and he plans to have his prostate-specific antigen measured shortly.

This study has 1 major limitation. It is a single case report from a single institution in Japan. Additional clinicopathological analyses, including multicenter studies, are needed to determine the cause and pathophysiology of this perplexing condition definitively. Making a definitive statement regarding prognosis based on this case alone is impossible. Therefore, reports from other countries, cultures, and hospitals are awaited.

## CONCLUSIONS

The authors experienced a rare and valuable case of a man with SC of the breast. As opportunities for genetic testing increase through companion diagnostics, cases of SC with *BRCA* pathogenic variants will likely accumulate. Although the prognosis of SC is unclear, it may become apparent in the future. Therefore, reports of SC with *BRCA* pathogenic variants worldwide are awaited.

## ACKNOWLEDGMENTS

The authors thank Mr. Naoki Ooishi, Mr. Takayoshi Hirota, and Mr. Kuniaki Muramatsu (Division of Pathology and Oral Pathology, and Department of the Diagnostic Pathology, Shimada General Medical Center, Shimada, Shizuoka, Japan) for performing immunohistochemical staining, and Prof. Hiroko Tina Tajima (St. Marianna University School of Medicine, Kawasaki, Kanagawa, Japan) for kindly reviewing and editing the English manuscript. The main focus of this paper was presented at the poster session of the 85th Annual Congress of the Japan Surgical Association, held on November 16, 2023, in Okayama, Japan.

## DECLARATIONS

### Funding

The authors declare that this research did not receive a specific grant from any public, commercial, or not-for-profit funding agency.

### Authors’ Contributions

Each author has participated sufficiently in the work to take public responsibility for appropriate portions of the content.

KT analyzed the radiological features.

MK, TI, YS, YY, TK, Ryosuke K, Ryohei K, KS, TW, TU, MN, and KK, the attending surgeon of the present case, earnestly discussed clinical problems.

MK and MT analyzed the histopathological features.

MK wrote and prepared the manuscript under the supervision of TI and MT.

HW is the senior author supervising the manuscript.

All authors read and approved the final manuscript for publication.

### Availability of data and materials

All data generated or analyzed during this study are included in this article. Further inquiries can be directed to the corresponding author.

### Ethics approval and consent to participate

Not applicable.

### Consent for publication

Written consent was obtained from the patient for the use of data and images and publication of this report.

### Competing interests

The authors declare that they have no competing interests.
